# Relationship between Postoperative Complications and Nutrition-Related Indices including Prealbumin in Postoperative Gastric Cancer Patients

**DOI:** 10.31662/jmaj.2025-0017

**Published:** 2025-06-27

**Authors:** Keisuke Morikawa, Hiroyuki Takemura, Kana Kitayama, Shogo Inaba, Haruka Imaoka, Yu Hashitsume, Yuta Suzuki, Kazuyuki Tabira

**Affiliations:** 1 Department of Rehabilitation, Matsusaka Municipal Hospital, Matsusaka, Japan; 2 Graduate School of Health Sciences, Kio University, Kitakatsuragi, Japan

**Keywords:** Controlling Nutrition Status, gastric cancer, postoperative complications, prealbumin

## Abstract

**Introduction:**

Several studies have reported an association between postoperative complications and nutritional indices. Among these, serum prealbumin (PAB) levels have recently attracted attention as an indicator of nutritional status. However, the nutritional index most strongly associated with postoperative complications remains unclear. This study aimed to investigate the relationship between postoperative complications and nutrition-related indices, including PAB, in patients who underwent surgery for gastric cancer.

**Methods:**

A total of 108 patients who underwent gastric cancer surgery were classified into 2 groups based on the presence or absence of postoperative complications. PAB, serum albumin (ALB), Controlling Nutritional Status (CONUT) score, and Prognostic Nutritional Index (PNI) were used as preoperative nutrition-related indices. The relationship between postoperative complications and nutrition-related indices was examined using multivariate logistic regression analyses.

**Results:**

Among the participants, 84 were classified into the uncomplicated group and 24 into the complicated group. Multivariate logistic regression analysis revealed significant associations between all nutrition-related indices and postoperative complications, including PAB (odds ratio [OR], 0.808; 95% confidence interval [CI]: 0.716-0.911, p = 0.001), CONUT score (OR, 1.338; 95% CI: 1.095-1.635, p = 0.004), ALB (OR: 0.364, 95% CI: 0.157-0.845, p = 0.019), and PNI (OR: 0.931, 95% CI: 0.870-0.997, p = 0.042). Among these, PAB showed the strongest association, followed by CONUT, ALB, and PNI.

**Conclusions:**

All nutritional indicators were associated with postoperative complications. Our findings suggest that PAB and CONUT scores may serve as useful predictors of postoperative complications in patients with gastric cancer.

## Introduction

Surgical treatment remains the fifth most common cancer in terms of incidence and overall mortality among all cancer patients ^(1)^. It is the mainstay of curative treatment for gastric cancer and is considered the most effective, especially when the disease is detected early ^(2)^. However, it is often associated with postoperative complications that are detrimental to the patient and significantly affect rehabilitation, including prolonged hospital stays, increased costs, decreased quality of life, and delayed initiation of subsequent postoperative adjuvant chemotherapy ^(3)^. Previous studies have reported that nutritional disorders are independently associated with the development of postoperative complications in esophageal, gastric, and other cancers, leading to delayed wound healing, impaired physical function, and poor prognosis ^(4)^. Additionally, nutrition-related indices, such as the Controlling Nutritional Status (CONUT) and Prognostic Nutritional Index (PNI), have been reported to be associated with postoperative complications ^(5), (6)^. However, these nutrition-related indices, including serum albumin (ALB), have long half-lives, making them less sensitive in detecting acute protein malnutrition, especially during the perioperative period ^(7)^. Recently, serum prealbumin (PAB) has gained attention as a protein for nutritional assessment due to its very short half-life of approximately 2 days, which may allow for a more accurate assessment of patient status in the acute care setting. It has been proposed as a useful nutritional biomarker for assessing patients at nutritional risk ^(8)^. We hypothesized that PAB, owing to its shorter half-life, may be a better predictor of postoperative complications in patients with gastric cancer than ALB. However, it remains unclear which nutritional indices are most strongly associated with postoperative complications. This study aimed to investigate the relationship between postoperative complications and nutrition-related indices, including PAB, in patients with gastric cancer.

## Materials and Methods

### Study design and subjects

**Figure 1.**
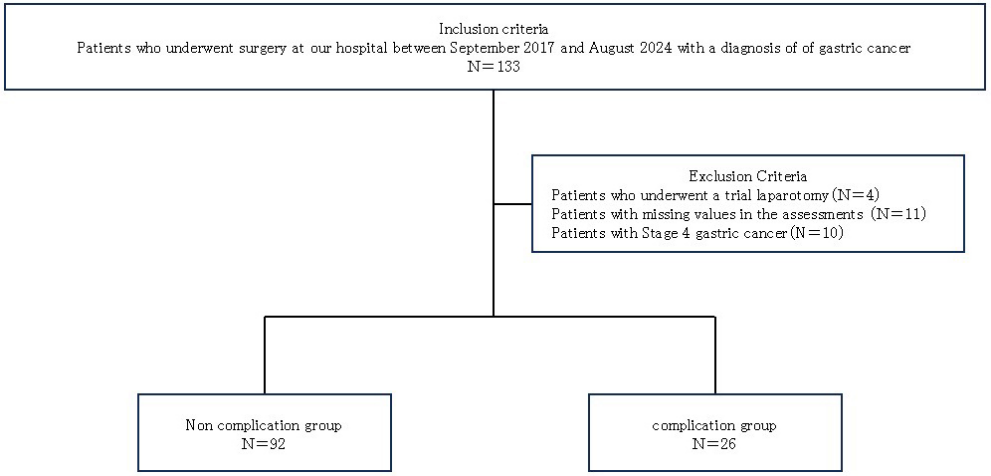
Flowchart of patient grouping.

This was a single-center, prospective observational study. This study was approved by the Ethics Review Committee of Matsusaka Municipal Hospital (J-54-170830-3-1) and conducted after obtaining written informed consent from all participants. The inclusion criterion was the presence of gastric cancer in 133 patients who had undergone surgery between September 2017 and August 2024 at our hospital. The exclusion criteria were as follows: patients without PAB measurement during preoperative assessment (N = 11), patients with stage 4 gastric cancer (N = 10), and patients who underwent trial laparotomy (N = 4). A total of 108 patients were included in the analysis (Figure 1). Patients were classified into 2 groups according to the presence or absence of postoperative complications. A postoperative complication was defined as grade 2 or higher according to the Clavien-Dindo classification, occurring within 30 days after surgery ^(9)^. Infectious complications were defined as wound complications, intraabdominal fluid retention or absence, anastomotic leak or fistula, traumatic pancreatitis, lung infection, urinary tract infection, cholangitis, sepsis, or systemic inflammatory response syndrome resulting from any of these conditions. Noninfectious complications included bleeding, bowel obstruction or ileus, anastomotic stenosis, and other complications affecting the circulatory, renal, or hepatic systems.

### Parameters

Patient background information included age, sex, body mass index, presence of comorbidities, and surgical details, such as cancer stage, site of resection (total or partial resection), surgical technique (open or laparoscopic), operation time, and blood loss. Pulmonary function tests were performed to measure vital capacity (%VC), forced VC (%FVC), forced expiratory volume in 1 second, and predicted forced expiratory volume in 1 second (%FEV1). Blood collection data, including carcinoembryonic antigen (CEA), carbohydrate antigen 19-9 (CA19-9), C-reactive protein (CRP), ALB, PAB, lymphocyte count, total cholesterol (TC), PNI, and CONUT, were calculated according to previous studies ^(5), (6)^. ALB, PAB, PNI, and CONUT were the nutrition-related indices used in this study. Pulmonary function tests and blood collection were performed one month before surgery.

PNI was calculated as 10 × serum ALB (g/dL) + 0.005 × total peripheral lymphocyte count (μL) ^(6)^. CONUT scores were calculated using serum ALB, TC concentrations, and total peripheral lymphocyte counts based on a previous report that used preoperative serum samples ^(10)^. ALB concentrations of ≥3.5, 3.0-3.49, 2.5-2.99, and <2.5 g/dL were scored 0, 2, 4, and 6 points, respectively. Total lymphocyte counts ≥1,600, 1,200-1,599, 800-1,199, and <800/mm^3^ were scored as 0, 1, 2, and 3 points, respectively. TC concentrations ≥180, 140-179, 100-139, and <100 mg/dL were scored as 0, 1, 2, and 3 points, respectively. The CONUT score was defined as the sum of the 3 components.

### Statistical analysis

Normality was assessed using the Shapiro-Wilk test, and comparisons between the 2 groups were performed using the Mann-Whitney U and χ^2^ tests. Multivariable logistic regression analyses were conducted to assess the association between postoperative complications and nutrition-related indicators, and odds ratio (OR) with 95% confidence intervals (CIs) were calculated. Both univariate and multivariate logistic regression analyses were performed with the presence or absence of postoperative complications as the dependent variable. Multivariate logistic regression analysis (forced entry method) was performed, adjusting for clinically important confounders (age, sex, surgical procedure, and %FEV1), with each nutrition-related indicator (PAB, ALB, PNI, and CONUT) included separately as an explanatory variable. Models 1, 2, 3, and 4 incorporated PAB, ALB, PNI, and CONUT, respectively. Age, sex, surgical procedure, and %FEV_1_ were used as independent variables because these factors may be associated with postoperative complications. All statistical analyses were performed using SPSS Statistics software (version 26.0; SPSS Inc., Chicago, IL, USA). Statistical significance was set at p < 0.05.

## Results

Participants were classified into 2 groups: 84 patients in the non-complication group and 24 patients in the complication group. Infectious complications were observed in 18 patients (75.0%), including 5 cases of suture failure, 4 cases of wound infection, 4 cases of respiratory infection, 2 cases of abscess, 2 cases of pancreatic leak, and one case of central venous catheter infection. Noninfectious complications were observed in 6 patients (25.0%), including 2 cases of ileus, one case of bowel obstruction, one case of acute heart failure, one case of subdural hematoma, and one case of hypoglycemia. The median time of blood collection was 20 days before surgery.

A comparison of patient backgrounds between the 2 groups is shown in Table 1. The complication group was older and had greater blood loss. Psychiatric disorders and respiratory diseases were more common in this group. Additionally, %VC and %FVC were significantly lower, while CEA and CRP were significantly higher, and TC was lower in the complication group. Regarding nutrition-related indices, ALB, PAB, and PNI were significantly lower in the complication group, whereas the CONUT score was significantly higher.

**Table 1.** Comparison of Patient Backgrounds between Two Groups.

	All	Non-complication	Complication	p value
	N=108	N=84	N=24	
Age, (y)	75 (67, 80)	73 (65, 80)	79 (73, 82)	0.039
Gender, (M/F）	82/26	63/21	19/5	0.791
BMI, (kg/m^2^)	22 (20, 24)	22 (20, 24)	22 (19, 24)	0.953
Comorbidity				
Psychiatric disorder, n (%)	16 (14.8)	7 (8.3)	9 (37.5)	0.001
Hypertension, n (%)	42 (38.9)	29 (34.5)	13 (54.2)	0.099
Diabetes, n (%)	29 (26.9)	22 (26.2)	7 (29.2)	0.797
Cerebrovascular, n (%)	8 (7.4)	6 (7.1)	2 (8.3)	1.000
Respiratory, n (%)	21 (19.4)	7 (8.3)	14 (58.3)	<0.001
Cardiovascular, n (%)	32 (29.6)	22 (26.2)	10 (41.7)	0.114
Surgical information				
Stage, (Ⅰ/Ⅱ/Ⅲ)	46/37/25	38/31/15	8/6/10	0.051
Location, (partial/total)	76/32	62/22	14/10	0.204
Surgical, (open/laparoscope)	81/27	61/23	20/4	0.423
Surgical duration, (min)	334 (295, 433)	346 (295, 433)	328 (296, 436)	0.503
Blood loss, (ml)	214 (75, 424)	173 (55, 339)	361 (140, 496)	0.041
Pulmonary function test				
%VC, (%)	89 (78, 101)	93 (83, 102)	82 (73, 90)	0.008
%FVC, (%)	90 (79, 102)	92 (83, 104)	81 (71, 85)	0.001
FEV_1_, (%)	73 (66, 82)	73 (67, 83)	72 (64, 81)	0.451
%FEV_1_, (%)	84 (68, 97)	86 (70, 102)	76 (64, 87)	0.107
Blood data				
CEA, (ng/mL)	2 (1, 4)	2 (1, 3)	3 (2, 6)	0.035
CA19-9, (U/mL)	5 (3,10)	5 (2,10)	4 (3,12)	0.814
CRP, (mg/L)	0.2 (0.1, 0.7)	0.2 (0.1, 0.4)	0.8 (0.1,1.7)	0.008
ALB, (g/dL)	3.8 (3.5,4.2)	3.9 (3.7,4.2)	3.5 (2.9,4.0)	0.001
PAB, (mg/dL)	21 (18, 26)	22 (19, 26)	18 (12, 20)	<0.001
LC, (/μL)	1536 (1110, 1961)	1560 (1200, 1961)	1314 (1030, 1973)	0.425
TC, (mg/dL)	187 (162, 204)	195 (169, 214)	152 (137, 175)	<0.001
PNI	46 (41, 51)	47 (43, 51)	42 (35, 48)	0.008
CONUT	1 (1, 3)	1 (0, 3)	4 (1, 6)	<0.001

Values are presented as the median (1st quartile, 3rd quartile) and number (%).

Table 2 shows the results of the univariate logistic regression analysis. Significant differences were observed for PAB, ALB, PNI, and CONUT.

**Table 2.** Factors Related to Postoperative Complications and Univariate and Multivariate Logistic Regression Analysis.

Variable	Univariate		
	OR	95%CI	p Value
Age	1.064	1.005-1.126	0.034
Female	1.267	0.421-3.813	0.674
Surgical (open)	0.530	0.164-1.719	0.291
%FEV_1_	0.987	0.966-1.008	0.208
PAB	0.841	0.763-0.927	<0.001
ALB	0.299	0.137-0.653	0.002
PNI	0.918	0.862-0.977	0.007
CONUT	1.371	1.140-1.648	0.001

The results of the multivariate logistic regression analysis for models 1, 2, 3, and 4 are shown in Table 3. Model 1 showed a significant association with PAB (OR, 0.808, 95% CI: 0.716-0.911, p = 0.001). Model 2 showed a significant association with ALB (OR, 0.364, 95% CI: 0.157-0.845, p = 0.019). Model 3 showed a significant association with PNI (OR, 0.931, 95% CI: 0.870-0.997, p = 0.042). Model 4 showed a significant association with CONUT (OR 1.338, 95% CI: 1.095-1.635, p = 0.004).

**Table 3.** Factors Related to Postoperative Complications and Multivariate Logistic Regression Analysis.

Variable	multivariate		
	OR	95%CI	p Value
Model 1			
Age	1.030	0.965-1.100	0.370
Female	3.533	0.786-16.06	0.100
Surgical (open)	0.525	0.140-1.961	0.338
%FEV_1_	0.988	0.963-1.013	0.331
PAB	0.808	0.716-0.911	0.001
Model 2			
Age	1.037	0.972-1.106	0.271
Female	1.426	0.395-5.152	0.588
Surgical (open)	0.473	0.130-1.725	0.257
%FEV_1_	0.990	0.967-1.014	0.398
ALB	0.364	0.157-0.845	0.019
Model 3			
Age	1.038	0.974-1.107	0.249
Female	1.377	0.388-4.889	0.432
Surgical (open)	0.440	0.123-1.569	0.206
%FEV_1_	0.989	0.966-1.012	0.336
PNI	0.931	0.870-0.997	0.042
Model 4			
Age	1.030	0.966-1.098	0.366
Female	1.211	0.320-4.584	0.778
Surgical (open)	0.397	0.105-1.500	0.173
%FEV_1_	0.987	0.962-1.011	0.283
CONUT	1.338	1.095-1.635	0.004

All nutrition-related indices were associated with postoperative complications, with PAB, CONUT, ALB, and PNI showing the strongest associations in that order.

## Discussion

This study examined the relationship between postoperative complications and nutrition-related indices, particularly PAB, in patients undergoing gastric cancer surgery. The results demonstrated that approximately 75% of postoperative complications were infection-related. Furthermore, all nutrition-related indices studied were associated with postoperative complications, with the PAB levels and CONUT scores most strongly associated with postoperative complications in patients with gastric cancer.

PAB is a protein synthesized in the liver and is primarily used as an indicator of nutritional status and inflammation. In postoperative patients, PAB reflects the quality of nutritional status, and low levels suggest malnutrition or the presence of inflammation. Specifically, a decrease in PAB indicates postoperative malnutrition and has been confirmed to increase the incidence of postoperative and wound infections ^(11)^. From these observations, we conclude that there is a recognized association between PAB and postoperative complications. A previous study reported that PAB predicts postoperative complications in patients with gastric cancer with a high inflammatory response ^(12)^. As in previous studies, we confirmed that PAB, as a nutrition-related index, is associated with postoperative complications in Japanese patients with gastric cancer. It was also reported that PAB, but not ALB, was a predictor of postoperative complications in patients with gastric cancer ^(13)^. The results of our study also suggest that PAB may be a better predictor of postoperative complications than ALB, possibly because PAB has a short half-life of 2 days, reflecting the most recent nutritional status. Therefore, PAB can be used to detect early nutritional disorders, accurately predicting postoperative complications better than ALB. Furthermore, in this study, multivariate logistic regression analysis using general postoperative complication factors and PAB as an independent variable revealed a connection between PAB and postoperative complications, indicating that nutritional status is more closely linked to postoperative complications.

Additionally, the CONUT score was significantly linked to postoperative complications. The CONUT score serves as an effective and straightforward screening tool for assessing immunonutritional status, calculated from ALB and TC levels and total lymphocyte counts. The ALB concentration is a widely used marker of nutritional status, and decreased ALB levels are often associated with systemic inflammation affecting hepatic anabolism and catabolism ^(14)^. Lymphocyte count is an important parameter of immune status. It plays a crucial role in the host’s anticancer immunity by inducing cytotoxicity and inhibiting the proliferation, invasion, and migration of tumor cells. A decrease in lymphocyte count is a risk factor for poor prognosis in patients with colorectal cancer ^(15)^. TC concentration is associated with the prognosis of patients with various cancers ^(16)^. Since tumor tissue can reduce plasma cholesterol concentration, changes in the cholesterol concentration reflect the tumor burden and nutritional status. Therefore, the CONUT score, which combines these measures, indicates nutritional status as well as systemic inflammatory and immune responses, making it a better predictor of postoperative complications than ALB alone or PNI.

These findings emphasize the value of PAB and CONUT as tools for preoperative risk assessment in patients with gastric cancer. Recognizing patients with low PAB or elevated CONUT scores prior to surgery could facilitate targeted preoperative strategies, including nutritional supplementation and immune function optimization, aimed at minimizing postoperative risks and complications. Further studies should also assess the effectiveness of interventions combining preoperative nutritional supplementation (e.g., high-protein supplements or enteral nutrition) and exercise for patients with low PAB or high CONUT.

### Limitations

First, the small sample size may lead to considerable variability in the findings. Moreover, PAB levels may be influenced by thyroid function, which was not assessed in this study. As a single-center study, caution should be exercised when generalizing the results. A multicenter study could improve the external validity of these findings. Finally, this study did not assess the impact of preoperative nutritional interventions. Future research should explore their efficacy, particularly for patients with low PAB or high CONUT scores.

### Conclusions

Our findings suggest that PAB and CONUT scores may serve as useful predictors of postoperative complications in gastric cancer patients. Future studies should also assess the effectiveness of interventions combining preoperative nutritional supplementation (e.g., high-protein supplements or enteral nutrition) and exercise therapy for patients with low PAB or high CONUT scores. However, given the small sample size, these findings should be interpreted with caution.

## Article Information

### Conflicts of Interest

None

### Acknowledgement

The authors would like to thank all the rehabilitation team members at Matsusaka Municipal Hospital and Tabira Laboratory, Graduate School of Kio University.

### Author Contributions

Keisuke Morikawa and Kazuyuki Tabira made substantial contributions to the conception of this work; Hiroyuki Takemura, Shogo Inaba, and Yuta Suzuki contributed significantly to the data analysis and interpretation; Keisuke Morikawa and Kazuyuki Tabira made significant contributions to the design of the study and the interpretation of the data; Keisuke Morikawa drafted the original manuscript.

All authors have approved the submitted version of the manuscript and agreed to be accountable for any part of the work.

### Approval by Institutional Review Board (IRB)

IRB Approval Code: J-54-170830-3-1

Institution: Ethics Committee of Matsusaka Municipal Hospital.
